# Novel Therapies in Diabetes: A Comprehensive Narrative Review of GLP-1 Receptor Agonists, SGLT2 Inhibitors, and Beyond

**DOI:** 10.7759/cureus.51151

**Published:** 2023-12-27

**Authors:** Olusegun A Olanrewaju, Fnu Sheeba, Avinash Kumar, Saad Ahmad, Narendar Blank, Reema Kumari, Komal Kumari, Tamara Salame, Ayesha Khalid, Nazdar yousef, Giustino Varrassi, Mahima Khatri, Satish Kumar, Tamam Mohamad

**Affiliations:** 1 Pure and Applied Biology, Ladoke Akintola University of Technology, Ogbomoso, NGA; 2 General Medicine, Stavropol State Medical University, Stavropol, RUS; 3 Medicine, Dow University of Health Sciences, Karachi, PAK; 4 Medicine, Bahria University Medical and Dental College, Karachi, PAK; 5 Medicine, Shalamar Medical and Dental College, Lahore, PAK; 6 Internal Medicine, Liaquat University of Medical and Health Sciences, Hyderabad, PAK; 7 Medicine, Jinnah Postgraduate Medical Centre, Karachi, PAK; 8 Medicine, New Medical Centre Royal Family Medical Centre, Abu Dhabi, ARE; 9 Biological Sciences, Wayne State University, Detroit, USA; 10 Medicine, Fatima Memorial College of Medicine and Dentistry, Lahore, PAK; 11 Medicine, University of Kalamoon, Deir Atiyah An-Nabek, SYR; 12 Pain Medicine, Paolo Procacci Foundation, Rome, ITA; 13 Internal Medicine/Cardiology, Dow University of Health Sciences, Civil Hospital Karachi, Karachi, PAK; 14 Medicine, Shaheed Mohtarma Benazir Bhutto Medical College, Karachi, PAK; 15 Cardiovascular Medicine, Wayne State University, Detroit, USA

**Keywords:** renal outcomes, cardiovascular protection, emerging therapies, sglt2 inhibitors, glp-1 receptor agonists, diabetes management

## Abstract

Diabetes mellitus, a widespread metabolic illness with increasing global occurrence, continues to have a significant impact on public health. Diabetes is a condition marked by long-term high blood sugar levels. It is caused by a combination of genetic, environmental, and lifestyle factors, which lead to problems with insulin production and insulin resistance. This dysfunctional state disturbs the delicate balance of glucose regulation, promoting the emergence of problems in both large and small blood vessels that have a substantial impact on illness and death rates. Traditional therapy methods have traditionally given more importance to managing blood sugar levels by using insulin sensitizers, secretagogues, and other medications that lower glucose levels. Advancements in our understanding of the underlying mechanisms of diabetes have led to a significant change in approach, focusing on comprehensive therapies that target not only high blood sugar levels but also the accompanying dangers to the heart and kidneys.

This study examines the evolving field of diabetes therapies, explicitly highlighting the significance of GLP-1 receptor agonists and SGLT2 inhibitors. These two types of drugs have become essential components in modern diabetes management. GLP-1 receptor agonists replicate the effects of natural glucagon-like peptide-1, leading to insulin production that is reliant on glucose levels, reducing the release of glucagon, and providing cardiovascular advantages that go beyond controlling blood sugar levels. SGLT2 inhibitors, however, act on the process of renal glucose reabsorption, leading to increased excretion of glucose in the urine and showing significant benefits for cardiovascular and renal protection. This extensive investigation seeks to contribute to the ongoing discourse on diabetes therapies by synthesizing existing research. This review aims to provide clinicians, researchers, and policymakers with a comprehensive understanding of the disease background and the specific pharmacological details of GLP-1 receptor agonists, SGLT2 inhibitors, and other related treatments. The goal is to assist them in developing more effective and personalized strategies to tackle the complex challenges presented by diabetes.

## Introduction and background

The widespread and increasing occurrence of diabetes mellitus on a global scale makes it a significant public health issue, requiring ongoing investigation and development of new treatment approaches. Diabetes is a condition marked by consistently high levels of glucose in the blood due to insufficient insulin secretion, impaired insulin function, or both. It involves a range of intricacies that go beyond its direct metabolic effects. This introduction provides a detailed overview of diabetes, starting with its background and then discussing the historical development of diabetic treatments. It concludes with a thorough examination of the need for new therapeutic approaches. The importance of diabetes in the global health context is emphasized by data from the International Diabetes Federation (IDF), which said that about 463 million individuals were affected worldwide in 2019, with forecasts predicting a remarkable rise to 700 million by 2045 [1].

This pandemic encompasses significant economic, social, and healthcare consequences, going beyond just numerical statistics. Diabetes is not just a single health problem but rather a condition that predicts significant illness and death. It is closely connected to major difficulties affecting large blood vessels, such as cardiovascular illnesses, as well as minor blood vessel disorders including retinopathy and nephropathy. The fundamental nature of diabetes pathogenesis involves intricate disturbances in glucose balance. The range of clinical symptoms, including Type 1 and Type 2 diabetes, represents the various causes and mechanisms that contribute to this intricate condition [2]. Regardless of whether it is defined by insulin resistance, decreased insulin secretion, or a combination of both, the resulting effects extend to other organ systems, intensifying the diversity of clinical manifestations and difficulties related to successful treatment.

The complexities of diabetes become more apparent when examining its molecular aspects, where hereditary and environmental variables interact to cause abnormalities in insulin signaling pathways. Diabetes etiology involves abnormalities in the functioning of pancreatic beta cells, insulin sensitivity in peripheral tissues, and the regulatory mechanisms that control glucose metabolism. To effectively treat diabetes, it is crucial to have a thorough grasp of its complexities that go beyond the visible clinical symptoms. This knowledge forms the basis for a more comprehensive and holistic approach to diabetes care [3]. The history of diabetes treatment has been marked by a continuous effort to develop effective therapies ever since the groundbreaking discovery of insulin by Banting and Best in 1921. In the following years, oral hypoglycemic medications were introduced in the 1950s, which was a significant turning point in the expansion of treatment choices. As comprehension increased, improvements were made to insulin formulations, and therapy approaches developed to include a range of interventions, from lifestyle adjustments to pharmacological substances [4].

Traditional treatment methods, such as insulin therapy, oral hypoglycemic medications, and lifestyle changes, have played a fundamental role in managing diabetes. Nevertheless, despite their historical importance, these methods still have inherent constraints. Attaining and sustaining ideal glucose control continues to be a chronic difficulty, frequently hindered by adverse reactions, patient non-adherence, and the complex interaction between diabetes and concurrent comorbidities. Therefore, the changing field of diabetic treatments requires a careful reassessment that goes beyond traditional frameworks. The constraints of standard methods for managing diabetes are not exclusively limited to regulating blood sugar levels. Diabetes, acknowledged as a systemic ailment with extensive consequences, requires a more comprehensive therapeutic approach. Cardiovascular disorders, which significantly contribute to the illness and death rates associated with diabetes, are not adequately targeted by conventional treatment methods. The presence of kidney-related issues adds to the difficulty, highlighting the importance of therapies that go beyond focusing solely on glucose levels [4].

The need for new therapeutic approaches in diabetes arises from a combination of reasons, indicating the inherent constraints of conventional methods and the advancing comprehension of diabetes as a complex condition. This paradigm change is defined by a shift away from solely prioritizing glycemic control to adopting a more comprehensive approach to care [4]. As the therapy of diabetes expands beyond focusing exclusively on glucose levels, it becomes crucial to incorporate cardiovascular and renal factors. Precision medicine is a guiding philosophy that directs treatment interventions toward personalized techniques that are specifically customized to the unique characteristics of each patient. The variability observed in the way diabetes presents highlights the significance of customizing therapies to match the various metabolic, genetic, and environmental components that contribute to the condition. Customized methods not only enhance the effectiveness of medical treatments but also reduce the negative consequences commonly linked to traditional therapies [5].

The pursuit of new treatments for diabetes is motivated by a deep recognition of the intricate nature of the disease and a sincere desire for more effective and focused interventions. Glucagon-like peptide-1 (GLP-1) receptor agonists have been identified as significant contributors among these new drugs, showcasing diverse impacts on glucose regulation, appetite control, and cardiovascular results. Sodium-glucose cotransporter 2 (SGLT2) inhibitors, by decreasing the reabsorption of glucose in the kidneys, have proven to be effective in controlling blood sugar levels and have also shown notable benefits in protecting the cardiovascular system and kidneys [5]. Ultimately, the development of diabetes treatments has progressed over time, starting with the fundamental discovery of insulin and advancing to the present period characterized by a more sophisticated comprehension of the disease's intricacies. The need for new therapeutic approaches in diabetes arises from the limitations of traditional methods, the necessity for comprehensive management, and the ongoing quest for precision medicine. The increasing discovery of innovative treatments marks the beginning of a new age in diabetes care, with the potential for better results and improved quality of life for individuals dealing with this prevalent metabolic condition.

## Review

Methodology

The main aim of this narrative review is to thoroughly examine the current body of research on innovative treatments for diabetes, mainly focusing on GLP-1 receptor agonists and SGLT2 inhibitors and developing therapeutic approaches. The review sought to consolidate previous research findings on the mechanisms by which these medicines operate, their clinical effectiveness, safety profiles, and potential synergistic effects. This aims to provide a nuanced comprehension of their contribution to advancing diabetes care. We performed an extensive literature search on prominent databases such as PubMed, Embase (Excerpta Medica Database), Scopus, and the Cochrane Library. The search methodology employed a fusion of medical subject headings (MeSH) phrases and keywords about diabetes, GLP-1 receptor agonists, SGLT2 inhibitors, and new therapeutics. The review encompassed English papers from the beginning of databases until the end of the search, focusing on contemporary research and reviews. A thematic synthesis methodology was utilized to logically arrange and present previous study findings. The discussion covered key themes like the processes by which something works, the results observed in clinical settings, the safety characteristics, and the rising trends. This provided a retrospective overview of the current understanding of the subject. This narrative review acknowledged some limitations. The intrinsic diversity in the designs and demographics of the research included may induce heterogeneity, which can affect the applicability of the findings. Moreover, the dependence on published literature might result in bias since favorable outcomes are more prone to publication. Since this narrative review relies on existing material, obtaining ethical approval was unnecessary. Nevertheless, ethical considerations were thoroughly addressed to guarantee data sources' secrecy and proper attribution. The intellectual contributions of the original writers were acknowledged through the use of appropriate referencing and citation methods.

Pathophysiology of diabetes

Diabetes mellitus is a multifaceted metabolic illness marked by persistent high blood sugar levels due to irregularities in the production or function of insulin. This part offers an in-depth examination of the pathophysiology of diabetes, clarifying the fundamental mechanisms that contribute to its development and advancement.

Overview of Diabetes Mechanisms

The pathophysiology of diabetes involves a complex interaction between genetic, environmental, and behavioral variables; the categorization of diabetes into Type 1 and Type 2 highlights apparent differences in their underlying causes. Type 1 diabetes is caused by an autoimmune attack that leads to the loss of pancreatic beta cells, resulting in a complete lack of insulin. From an etiological perspective, this process typically begins at a young age and is impacted by genetic predisposition and environmental triggers, such as viral infections [6]. Type 2 diabetes, on the other hand, mainly arises from a combination of insulin resistance and decreased insulin production. Insulin resistance occurs when vital tissues that respond to insulin, such as muscle, liver, and adipose tissue, become less receptive. This hinders glucose absorption and results in higher glucose levels in the blood. At the same time, when confronted with higher demand, pancreatic beta cells cannot produce enough insulin to maintain normal blood sugar levels [6].

Genetic variables are crucial in determining the likelihood of diabetes, as specific gene variants are linked to Type 1 and Type 2 diabetes. Environmental factors, such as dietary choices, level of physical activity, and exposure to harmful substances, have a crucial role in the development of diabetes. To comprehend the mechanisms that drive diabetes, one must acknowledge the complex interplay between the immune system, pancreatic function, and environmental factors. Immunogenetic variables, such as the existence of particular human leukocyte antigen (HLA) genotypes, have a role in the autoimmune reaction in Type 1 diabetes. This autoimmune attack causes the targeted killing of pancreatic beta cells, significantly reducing insulin production [7]. In the context of Type 2 diabetes, the primary component is insulin resistance, which is influenced by hereditary variables contributing to an individual's susceptibility to this resistance. Particular genetic polymorphisms linked to insulin signaling pathways and glucose metabolism have a role in the differences in insulin sensitivity observed among individuals.

Furthermore, the interaction between genetics and the environment becomes apparent when considering obesity, a significant contributing factor to the development of Type 2 diabetes. Genetic factors and lifestyle decisions both have a role in the malfunction of fatty tissue, dramatically contributing to the development of insulin resistance. Understanding the genetic and environmental factors contributing to diabetes is crucial for developing personalized medicine strategies and targeted interventions based on an individual's risk profile [7].

Impaired Insulin Secretion and Insulin Resistance

Insulin resistance is a characteristic feature of Type 2 diabetes, a condition in which tissues that usually respond to insulin have decreased sensitivity, leading to a decrease in glucose uptake. Insulin's effects on essential areas such as skeletal muscle, liver, and adipose tissue are weakened, resulting in reduced glucose absorption by cells. The molecular mechanisms that cause insulin resistance entail disturbances in intracellular signaling pathways, leading to decreased glucose transporters' movement and impaired glucose utilization by cells. In obesity, the persistent increase in free fatty acids worsens insulin resistance by triggering inflammation and disrupting insulin signaling pathways. The dysfunctional adipose tissue exacerbates insulin resistance by releasing proinflammatory adipokines [7]. To comprehend the complex molecular mechanisms underlying insulin resistance, one must analyze the signaling channels that regulate glucose balance in the body. The insulin receptor, a protein embedded in the cell membrane of target cells, functions as the primary mediator of insulin's effects. Insulin attaching to its receptor triggers a series of internal processes, causing the movement of glucose transporters (GLUT4) to the cell membrane, enabling glucose uptake. Insulin-resistant states lead to abnormalities at multiple sites in this signaling cascade. The dysregulation of insulin receptor phosphorylation, defective activation of downstream signaling molecules such as insulin receptor substrate (IRS-1), and abnormal translocation of GLUT4, all contribute to decreased cellular responsiveness to insulin [8].

Moreover, inflammation, which is frequently linked to obesity, exacerbates insulin signaling impairment by inducing serine phosphorylation of IRS-1, resulting in the development of insulin resistance. Simultaneously, the malfunction of beta cells is crucial in advancing Type 2 diabetes. As insulin resistance increases, beta cells try to compensate by increasing insulin output. Nevertheless, continuous pressures on these cells can result in their depletion, leading to a relative insufficiency of insulin. Multiple factors contribute to the failure of beta cells, including genetic susceptibility, oxidative stress, and prolonged exposure to high blood sugar levels [8]. Glucotoxicity refers to the condition in which long-term high blood sugar levels damage the functioning of beta cells and increase cell death, worsening beta-cell malfunction. The malfunctioning of beta-cells entails complex molecular mechanisms occurring within the pancreatic islets.

The insulin synthesis process initiates with the insulin gene (INS) transcription to generate preproinsulin. The initial form of this substance undergoes enzymatic breakdown, resulting in the production of proinsulin, which is then divided to produce physiologically active insulin. Prolonged exposure to high glucose levels has harmful consequences on beta cells. Glucotoxicity causes endoplasmic reticulum stress, disturbs calcium balance, and stimulates the generation of reactive oxygen species (ROS), all contributing to the malfunction and death of beta cells [7,8]. In addition, the persistent rise of free fatty acids leads to lipotoxicity, further hampering beta-cell activity. Accumulation of lipids within cells, including ceramides and diacylglycerols, hinders the routes through which insulin signals and worsens oxidative stress, creating a harmful environment within the pancreatic islets. Understanding the complexities associated with beta-cell malfunction is essential to devise strategies to safeguard and improve beta-cell health. Promising approaches to address beta-cell dysfunction in diabetes include strategies to reduce endoplasmic reticulum stress, regulate lipid metabolism, and protect against oxidative stress [8,9].

Macrovascular and Microvascular Complications

Macrovascular complications: Diabetes significantly increases the likelihood of macrovascular problems, primarily cardiovascular illnesses. Diabetes is characterized by the accelerated development of atherosclerosis, which involves plaque accumulation in major arteries. Hyperglycemia exacerbates endothelial dysfunction, inflammation, and oxidative stress, creating an environment that promotes the development of atherosclerosis. Diabetic individuals are especially prone to developing coronary artery disease, peripheral arterial disease, and cerebrovascular accidents. The relationship between diabetes and cardiovascular illnesses is complex, encompassing dyslipidemia, hypertension, and the proinflammatory state associated with diabetes [9]. To comprehend the complex connection between diabetes and cardiovascular illnesses, it is essential to closely analyze the molecular and cellular mechanisms that contribute to atherosclerosis. Endothelial dysfunction, marked by reduced ability of blood vessels to dilate and increased leakage of blood vessels, is a crucial initial occurrence in the development of atherosclerosis. Hyperglycemia exacerbates endothelial dysfunction through multiple pathways. Prolonged exposure to high glucose levels increases the synthesis of advanced glycation end-products (AGEs). This, in turn, causes oxidative stress and inflammation in the vascular endothelium.

In addition, hyperglycemia stimulates the activation of protein kinase C (PKC) and the hexosamine pathway, which worsens endothelial dysfunction. Endothelial dysfunction creates the conditions for attracting inflammatory cells, specifically monocytes, to the blood vessel wall. The monocytes undergo differentiation into macrophages, which initiate the process of foam cell production by absorbing oxidized low-density lipoprotein (LDL) particles. Foam cells gathering in the artery wall result in the development of fatty streaks, which are an early stage of atherosclerotic plaques [10]. Oxidative stress is a key factor in the development of atherosclerosis, where ROS produced in the blood vessel wall contribute to the oxidation of lipids and the promotion of inflammation. In addition, initiating proinflammatory signaling pathways, such as nuclear factor-kappa B (NF-κB), enhances the inflammatory response and stimulates the production of adhesion molecules, which aid in attracting immune cells to the atherosclerotic lesion. Gaining insight into the molecular mechanisms behind atherosclerosis in diabetes is crucial for developing precise treatment strategies. Strategies that aim to reduce oxidative stress, regulate inflammation, and maintain the health of blood vessel linings can potentially prevent and treat cardiovascular problems in people with diabetes [10].

Microvascular complications: Microvascular problems in diabetes result from the impairment of small blood arteries, leading to a large increase in morbidity. Retinopathy, nephropathy, and neuropathy are prevalent microvascular consequences. Retinopathy, a prominent factor in blindness, is distinguished by the harm inflicted upon the blood vessels in the retina. Long-term elevated blood sugar levels and high blood pressure cause damage to the small blood vessels of the retina, resulting in vision impairment. Nephropathy, often known as diabetic kidney disease, occurs due to injury to the small blood vessels in the kidneys. Glomerular damage, which causes proteinuria and gradual renal failure, results from hyperglycemia, inflammation, and hemodynamic variables. Neuropathy, which impacts both the peripheral and autonomic nerve systems, presents as pain, numbness, and gastrointestinal disruptions. The combination of microvascular alterations and oxidative stress plays a role in causing nerve injury and impairing neuronal function [11].

The development of microvascular problems in diabetes is closely associated with the combined impact of long-term high blood sugar levels, abnormal lipid levels, and high blood pressure. The development of microvascular problems is driven by common factors, such as oxidative stress, inflammation, and the creation of advanced glycation end-products (AGEs). Diabetic retinopathy occurs when high blood sugar levels cause oxidative stress in the retina's small blood vessels, resulting in the generation of ROS and the initiation of proinflammatory pathways. Elevated expression of vascular endothelial growth factor (VEGF) additionally contributes to the formation of abnormal blood vessels in the retina, disturbing regular vision. Diabetic nephropathy is defined by damage to the glomeruli and the gradual deterioration of kidney function. Hyperglycemia triggers the production of AGEs, which build up in the glomeruli, leading to inflammation and fibrosis. In addition, dyslipidemia plays a role in the accumulation of lipids in the blood vessels of the kidneys, which worsens renal injury [10,11]. Neuropathy, which impacts both sensory and autonomic nerves, is caused by a combination of metabolic, vascular, and immunologic causes. Hyperglycemia triggers oxidative stress and inflammation, which in turn cause nerve damage, affecting neuronal function and resulting in the distinctive symptoms of neuropathy. Comprehending the common pathways that support microvascular problems is essential for creating therapies that tackle the complex nature of these complications. Therapeutic approaches that target oxidative stress reduction, inflammation regulation, and vascular health maintenance provide opportunities for avoiding and treating microvascular problems in diabetes [11].

Conventional therapeutic approaches

Insulin Sensitizers

Insulin sensitizers are fundamental to treating Type 2 diabetes mellitus (T2DM). Their primary purpose is to address insulin resistance and improve glucose absorption by tissues in the body's periphery. Thiazolidinediones (TZDs), including pioglitazone and rosiglitazone, are commonly used insulin sensitizers. These medications stimulate the peroxisome proliferator-activated receptor (PPAR) gamma (γ) (PPAR-γ), enhancing insulin sensitivity in adipose tissue, skeletal muscles, and the liver. Pioglitazone, belonging to the TZD class, has shown effectiveness in improving insulin sensitivity and regulating glycemic control. Research has demonstrated that pioglitazone decreases glucose production in the liver, improves glucose absorption in the peripheral tissues, and reduces inflammation linked to insulin resistance [11]. Nevertheless, the apprehensions regarding possible adverse effects, such as weight gain and elevated susceptibility to heart failure, require meticulous evaluation before administering this medication. Rosiglitazone, a different TZD, has been the subject of criticism because of its connection to a higher likelihood of cardiovascular events. Although it has powerful insulin-sensitizing effects, concerns regarding its impact on cardiovascular health have resulted in limitations on its usage in certain areas [11]. This dispute highlights the significance of continuous research and monitoring in assessing the advantages and disadvantages of insulin sensitizers in managing T2DM.

Secretagogues

Secretagogues enhance insulin release from pancreatic beta cells, targeting the reduced insulin secretion aspect of T2DM. Sulfonylureas, including glyburide, glipizide, and glimepiride, are well-established secretagogues crucial in treating T2DM. Sulfonylureas exert their effects by attaching to the sulfonylurea receptor on beta cells, releasing insulin. These medications have been extensively utilized for many years, effectively reducing blood glucose levels. Nevertheless, worries over hypoglycemia and decreasing effectiveness over time have led to a reassessment of their function in managing T2DM [12]. Furthermore, the influence of these factors on cardiovascular outcomes is still being actively studied.

Challenges and Limitations

Insulin sensitizers and secretagogues have been influential in managing T2DM. However, several obstacles and limitations are associated with their use, which require a careful and detailed approach. An impediment to the utilization of insulin sensitizers and secretagogues is the variability in the therapeutic response observed among patients with T2DM. Genetic factors, lifestyle choices, and the length of time a person has had diabetes, all play a role in the differences observed in the effectiveness of treatments [12]. Customizing treatment approaches according to specific patient attributes is crucial for maximizing the effectiveness of therapy. Both insulin sensitizers and secretagogues are linked to a range of undesirable consequences. Insulin sensitizers such as TZDs can cause fluid retention, edema, and weight gain. Conversely, sulfonylureas provide a heightened susceptibility to hypoglycemia, especially in older patients or those with impaired kidney function [12]. We must carefully weigh the advantages of glycemic control against the possible drawbacks of adverse effects while making clinical decisions.

T2DM is a disorder that worsens over time, and the effectiveness of medications that improve insulin sensitivity and stimulate insulin secretion may decrease as the activity of beta cells deteriorates [13]. The need for a comprehensive and adaptable treatment approach is emphasized by this progressive nature, which may include the use of combination therapies, lifestyle changes, and timely revisions in pharmaceutical regimens. Ultimately, traditional therapy methods for T2DM, such as insulin sensitizers and secretagogues, have effectively addressed the intricate interaction between insulin resistance and decreased insulin secretion. The medicine classes of pioglitazone and sulfonylureas demonstrate the effectiveness of these medications, although their utilization is full of difficulties and constraints. The presence of negative consequences, variation in how individuals respond to cure, and the gradual development of T2DM make it necessary to adopt a careful and personalized approach to treatment. Continual research and clinical awareness are crucial as we navigate the management of T2DM. Advancements in pharmacology and an enhanced comprehension of the molecular and genetic foundations of T2DM offer the potential for improved and tailored therapeutic approaches. To overcome the problems and limits, the medical community can further progress and enhance outcomes for persons with T2DM.

GLP-1 receptor agonists: a comprehensive analysis

The management of T2DM has shown notable progress, with various therapeutic choices designed to target the intricate underlying mechanisms of the disease. Out of all these options, GLP-1 receptor agonists have become recognized as a unique and encouraging group of substances. This essay thoroughly examines GLP-1 receptor agonists, delving into their mechanism of action, clinical effectiveness in regulating blood sugar levels, cardiovascular advantages, safety characteristics, and a comparative evaluation with traditional treatments.

Mechanism of Action

GLP-1 receptor agonists imitate the natural incretin hormone GLP-1, which is discharged from the intestine when nutrients are consumed. These agonists exert their effects by activating the GLP-1 receptor, which is highly expressed in pancreatic beta cells and other tissues. The primary function of GLP-1 receptor agonists is to increase insulin release in response to glucose levels. This mechanism guarantees that insulin release is stimulated when blood glucose levels are high, reducing the likelihood of hypoglycemia [14]. GLP-1 receptor agonists inhibit the secretion of glucagon, the hormone that promotes elevated blood glucose levels. This simultaneous effect on insulin and glucagon helps to enhance the regulation of glucose levels after a meal. GLP-1 receptor agonists also can decelerate stomach emptying, decreasing the speed at which nutrients are absorbed. The delay after a meal contributes to better management of blood sugar levels and a feeling of fullness [14]. Clinical studies have demonstrated the effectiveness of this delay in controlling blood sugar levels. GLP-1 receptor agonists have shown impressive effectiveness in attaining and sustaining glycemic control in persons with T2DM. GLP-1 receptor agonists have been studied in clinical trials as a standalone treatment and in conjunction with other antidiabetic medications. Utilizing them as the sole remedy has demonstrated notable decreases in glycated hemoglobin (HbA1c) levels, and they have been validated as being efficacious when used in conjunction with metformin, sulfonylureas, and insulin [15]. GLP-1 receptor agonists are notable for their connection with weight loss, making them especially appealing in treating T2DM, a condition frequently accompanied by obesity. The weight loss can be related to a decrease in appetite and a reduction in the amount of food consumed. Research has indicated that GLP-1 receptor agonists maintain their ability to regulate blood sugar levels over an extended period while positively impacting beta cells' function. The longevity of treatment is an essential factor to consider while managing T2DM, as it is associated with a gradual decrease in beta cell activity [15].

Beyond Glycemic Control: Cardiovascular Benefits

GLP-1 receptor agonists have been increasingly acknowledged for their positive impact on cardiovascular health in recent years. Significant cardiovascular trials, such as LEADER (Liraglutide Effect and Action in Diabetes: Evaluation of Cardiovascular Outcome Results) and SUSTAIN-6 (Trial to Evaluate Cardiovascular and Other Long-term Outcomes With Semaglutide in Subjects With Type 2 Diabetes), have shown that GLP-1 receptor agonists can effectively decrease major adverse cardiovascular events, such as cardiovascular death, nonfatal myocardial infarction, and nonfatal stroke, in patients with Type 2 diabetes. The cardiovascular advantages of GLP-1 receptor agonists are complex and include multiple processes. These benefits may encompass endothelial function enhancements, decreased arterial stiffness, anti-inflammatory properties, and even direct effects on the heart. It is crucial to evaluate the safety and tolerability of GLP-1 receptor agonists, notwithstanding their favorable glycemic and cardiovascular characteristics. Frequent adverse effects encompass nausea, emesis, and diarrhea, particularly during the initial dose adjustment phase. The gastrointestinal effects often have a short duration and decrease gradually as time passes. GLP-1 receptor agonists have aroused concerns over the potential for pancreatitis and pancreatic cancer. Nevertheless, the research substantiating these concerns remains equivocal, and regulatory bodies must establish a definitive correlation. Reports have indicated that immunogenicity can occur, resulting in the production of antibodies against GLP-1 receptor agonists. Nevertheless, the precise clinical consequences of immunogenicity remain unclear, and severe allergic reactions are infrequent [15].

Comparative Evaluation of Conventional Therapies

A comparative analysis of GLP-1 receptor agonists and conventional therapy is necessary to understand the significance of GLP-1 receptor agonists in managing T2DM. GLP-1 receptor agonists have several advantages over insulin sensitizers like TZDs, including weight loss, reduced risk of hypoglycemia, and possibly cardiovascular benefits. Nevertheless, apprehensions regarding gastrointestinal adverse effects can impact the selection among these categories. GLP-1 receptor agonists have comparable glycemic effectiveness to sulfonylureas while providing weight reduction advantages and a reduced likelihood of hypoglycemia. The selection of these groups may vary based on particular patient attributes, such as obesity and susceptibility to hypoglycemia [15]. The choice between GLP-1 receptor agonists and other types of antidiabetic drugs for combination therapy is determined by each patient's specific requirements and preferences. Combination regimens aim to maximize glycemic control while minimizing side effects and addressing any concurrent medical conditions. GLP-1 receptor agonists have become a valuable treatment choice for managing T2DM, effectively controlling blood sugar levels, offering cardiovascular advantages, and promoting weight loss. Due to their distinctive method of operation, positive safety record, and ability to be tolerated well, they are an appealing option for a wide variety of patients. As our knowledge of T2DM and its treatment options improves, GLP-1 receptor agonists emerge as evidence of the advancements made in customizing medicines to tackle the complex character of this chronic disorder. Subsequent investigations should conduct more extensive studies on the prolonged cardiovascular impacts of GLP-1 receptor agonists, provide a clearer understanding of their modes of operation, and investigate potential collaborations with other therapeutic approaches. The incorporation of GLP-1 receptor agonists into personalized treatment algorithms shows potential for improving outcomes and boosting the overall quality of life for persons with T2DM as the area of diabetes management progresses.

SGLT2 inhibitors: unveiling cardiovascular and renal protective effects

SGLT2 inhibitors have been recognized as a groundbreaking category of antidiabetic drugs, regulating blood sugar levels and exhibiting unparalleled cardiovascular and renal protective properties. This essay examines the various elements of SGLT2 inhibitors, including their mechanism of action, ability to increase glucose excretion, impact on cardiovascular health, renal protective effects, and a comparative analysis with GLP-1 receptor agonists.

Mechanism of Action: Renal Glucose Reabsorption Inhibition

The core of the mechanism of SGLT2 inhibitors is the suppression of renal glucose reabsorption. The proximal renal tubules are crucial in glucose reabsorption from the glomerular filtrate. Most of this reabsorption is carried out by SGLT2, which is found in the proximal convoluted tubule [16]. SGLT2 inhibitors, including dapagliflozin, canagliflozin, and empagliflozin, specifically block the action of SGLT2, resulting in a higher amount of glucose being excreted in the urine. These medicines reduce blood glucose levels by inhibiting glucose reabsorption, regardless of insulin activity. This makes them desirable for people with T2DM. SGLT2 inhibitors decrease hyperglycemia and facilitate caloric loss, leading to weight reduction through the glycosuric effect. In addition, the osmotic diuretic impact causes natriuresis, which helps lower blood pressure [16]. The diverse effects of SGLT2 inhibitors create an opportunity to investigate the cardiovascular and renal advantages they offer. Multiple clinical trials have repeatedly shown that SGLT2 inhibitors effectively reduce HbA1c levels. EMPA-REG OUTCOME (Empagliflozin Cardiovascular Outcome Event Trial in T2DM Patients) and DECLARE-TIMI 58 (Dapagliflozin Effect on Cardiovascular Events-Thrombolysis in Myocardial Infarction 58) are two studies that have demonstrated notable decreases in HbA1c levels in patients with T2DM. Specifically, empagliflozin and dapagliflozin effectively reduce HbA1c, as indicated by these studies [16]. The weight-reducing properties of SGLT2 inhibitors have implications for factors that increase the risk of cardiovascular disease. Decreased body weight is linked to insulin sensitivity, lipid profiles, and blood pressure enhancements. These factors contribute to the overall cardiovascular advantage found in clinical studies.

Cardiovascular Outcomes

Significant cardiovascular outcome trials, such as EMPA-REG OUTCOME and CANVAS (Canagliflozin Cardiovascular Assessment Study), have presented convincing proof of the cardiovascular advantages linked to SGLT2 inhibitors. The tests showed a notable decrease in major adverse cardiovascular events, such as cardiovascular death, nonfatal myocardial infarction, and nonfatal stroke, when using empagliflozin and canagliflozin, respectively [17]. SGLT2 inhibitors have shown significant improvements in the treatment of heart failure. Clinical trials like EMPEROR-Reduced (Empagliflozin Outcome Trial in Patients With Chronic Heart Failure With Reduced Ejection Fraction) and DAPA-HF (Dapagliflozin and Prevention of Adverse Outcomes in Heart Failure) have shown a decrease in hospitalizations due to heart failure and cardiovascular mortality in patients, regardless of whether they have diabetes or not. The specific mechanisms that explain the cardiovascular advantages of SGLT2 inhibitors are still being actively investigated. Suggested tools are enhanced myocardial energetics, decreased arterial stiffness, and regulation of neurohormonal pathways, such as the renin-angiotensin-aldosterone system [17].

Renal Protection

SGLT2 inhibitors have shown impressive renal protective effects and cardiovascular advantages. Albuminuria is a widely recognized indicator of impaired kidney function and increased risk of cardiovascular problems. The CREDENCE (Canagliflozin and Renal Events in Diabetes With Established Nephropathy Clinical Evaluation) and EMPA-KIDNEY (Study of Heart and Kidney Protection with Empagliflozin) clinical trials demonstrated a notable decrease in albuminuria when using canagliflozin and empagliflozin, respectively [17]. SGLT2 inhibitors have been linked to a deceleration of chronic kidney disease (CKD) advancement, as demonstrated by a decrease in the rate at which blood creatinine doubles and the necessity for renal replacement therapy [18]. The renoprotective effects go beyond regulating blood sugar levels, suggesting a direct influence on renal pathways.

Comparative Analysis with GLP-1 Receptor Agonists

To fully comprehend the changing field of antidiabetic treatments, it is crucial to compare the cardiovascular and renal impacts of SGLT2 inhibitors with those of GLP-1 receptor agonists. Although SGLT2 inhibitors and GLP-1 receptor agonists provide cardiovascular advantages, they achieve these effects through distinct pathways. SGLT2 inhibitors have consistently demonstrated a decrease in occurrences of heart failure events and significant adverse cardiovascular events [18]. GLP-1 receptor agonists, as previously mentioned, mainly provide cardiovascular advantages by enhancing endothelial function, reducing arterial stiffness, and exerting anti-inflammatory actions. SGLT2 inhibitors demonstrate substantial renal protective benefits, specifically in decreasing albuminuria and decelerating the course of CKD. Although GLP-1 receptor agonists provide certain advantages for the kidneys, they do not have the same level of impact as SGLT2 inhibitors [18]. These classes' different methods of action probably contribute to their diverse effects on renal outcomes. SGLT2 inhibitors and GLP-1 receptor agonists are linked to weight reduction, rendering them beneficial in managing obesity, prevalent comorbidity in T2DM. SGLT2 inhibitors promote weight reduction by increasing urinary glucose excretion, while GLP-1 receptor agonists decrease hunger and reduce food consumption [18]. The effectiveness of glycemic management, as determined by the decrease in HbA1c levels, is similar in both classes.

SGLT2 inhibitors have revolutionized the field of T2DM treatment by not only efficiently reducing blood glucose levels but also exhibiting notable cardiovascular and renal protective properties. Their mode of operation, which involves blocking glucose reabsorption in the kidneys, leads to a range of advantages, such as promoting glucose excretion, improving cardiovascular health, and protecting the kidneys. SGLT2 inhibitors have become a fundamental aspect of managing T2DM, particularly in patients with existing cardiovascular disease or heart failure, due to their significant ability to reduce major adverse cardiovascular events and heart failure events. The renal protective effects, explicitly targeting the reduction of albuminuria and the deceleration of CKD progression, represent a significant change in how we approach the complex relationship between diabetes and renal problems. As we further understand the intricacies of managing diabetes, SGLT2 inhibitors demonstrate the ability of antidiabetic treatments to go beyond regulating blood sugar levels. The comparison of GLP-1 receptor agonists highlights the distinct advantages and diverse ways they work in the growing range of diabetic drugs. Future research efforts show potential for better understanding the molecular mechanisms that support the cardiovascular and renal advantages of SGLT2 inhibitors, leading to ongoing progress in diabetes treatment.

Emerging paradigms in diabetes management

The field of diabetes care is constantly changing, characterized by innovative developments in treatment tactics that go beyond conventional methods. This essay examines new approaches to managing diabetes, explicitly looking at novel medicines beyond GLP-1 receptor agonists and SGLT2 inhibitors. Furthermore, it explores the molecular targets and signaling pathways that form the basis of these innovative therapeutics and examines the possibility of personalized methods using precision medicine.

Innovative Therapies Beyond GLP-1 and SGLT2

GLP-1 receptor agonists have shown remarkable effectiveness in controlling blood sugar levels and improving cardiovascular outcomes. However, new therapeutic approaches are now emerging in the field, which will provide more options for managing T2DM. Tirzepatide, a type of dual incretin receptor agonist, shows great potential in treating diabetes. These drugs stimulate GLP-1 and glucose-dependent insulinotropic polypeptide (GIP) receptors, resulting in a double incretin action. The SURPASS program and other clinical trials have demonstrated significant decreases in HbA1c levels and body weight, highlighting the potential of dual incretin receptor agonists as a holistic treatment choice [19]. Alternative approaches to PPAR agonists include selective PPAR-γ modulators (SPPARMs). Tiraglitazar, a compound that activates both PPAR alpha (PPAR-α) and PPAR-γ receptors, can enhance the body's response to insulin and regulate the breakdown and utilization of lipids. Preliminary research indicates potential advantages in regulating blood sugar levels without the negative consequences linked to specific conventional PPAR-γ agonists [19]. Further investigation into PPAR agonists may provide alternative treatment options for people with T2DM. The comprehension of the molecular foundations of diabetes has facilitated the discovery of new targets and signaling pathways. Investigating these molecular pathways is crucial for developing precise and efficient treatments. G protein-coupled receptor 119 (GPR119) agonists have attracted interest due to their potential in regulating glucose balance. These drugs elicit incretin release and augment insulin secretion, providing a way to increase glycemic control. Despite initial positive findings in early clinical studies, additional study is required to comprehensively understand the clinical effectiveness and safety characteristics of GPR119 agonists [20].

Investigations have been conducted on mitochondrial modulators as prospective therapeutic targets due to the significance of mitochondrial dysfunction in the development of T2DM. Compounds that improve mitochondria functioning and decrease the harmful effects of oxidative stress can alleviate insulin resistance. Ongoing clinical trials are investigating the effectiveness and safety of mitochondrial modulators, which offer new possibilities for therapies that address the fundamental metabolic problems linked to T2DM [20]. As our knowledge of the variations in the underlying causes of diabetes increases, tailoring treatments based on individual characteristics becomes more critical in precision medicine. Customizing therapies according to individual attributes, such as genetic, metabolic, and lifestyle factors, can enhance treatment results and reduce negative consequences. Genomic advancements enable the detection of genetic variations linked to susceptibility to diabetes and the response to therapy. Incorporating genetic profiling into clinical practice allows for categorizing risk and pinpointing individuals who could benefit from targeted treatments. Genetic variations linked to a positive reaction to specific antidiabetic medications, including metformin, can guide treatment choices [20]. Metabolomic profiling provides a thorough examination of small-molecule metabolites in biological samples. Metabolomics offers valuable insights into disease causes and potential treatment targets by characterizing the metabolic fingerprint of individuals with diabetes. Metabolomic studies can identify biomarkers to be used as indicators of therapy response and help customize therapies based on individual metabolic patterns. Incorporating artificial intelligence (AI) and predictive modeling into diabetes care shows significant potential. Machine learning algorithms can analyze extensive datasets, encompassing clinical, genetic, and lifestyle information, to forecast the advancement of diseases and anticipate how patients will respond to treatment. This method enables the creation of customized treatment strategies, enabling doctors to predict personalized therapy requirements and modify interventions as time progresses [20].

Challenges and Considerations

As we begin implementing these novel strategies in diabetes care, addressing several obstacles and factors that require careful consideration is essential. The enduring safety of new medicines, apart from GLP-1 and SGLT2 inhibitors, is a significant concern. Thorough post-marketing surveillance and prolonged follow-up in clinical trials are necessary to evaluate the possibility of adverse events and unexpected outcomes over lengthy durations. The implementation of innovative treatments frequently prompts inquiries regarding their accessibility and pricing. It is crucial to ensure that novel therapies are accessible to a wide range of patients, especially those with few financial resources. Effective collaboration among pharmaceutical companies, healthcare providers, and politicians is crucial to tackle these difficulties and ensure fair and equal access to advanced therapies [21]. The interaction of several molecular pathways in the pathogenesis of diabetes adds to the intricacy of using combination treatments. Comprehending the interactions between various medicines and determining whether their effects are synergistic or antagonistic necessitates meticulous analysis. Combining medication with complementary mechanisms may improve effectiveness while reducing negative side effects. The emergence of precision medicine and genetic profiling gives rise to ethical concerns about privacy, consent, and potential societal ramifications. It is essential to guarantee that persons comprehend the consequences of genetic testing and possess the autonomy to create well-informed judgments.

Furthermore, it is crucial to tackle any discrepancies in the availability of individualized medical treatments to avoid worsening current gaps in health. The future of diabetes management lies in the quest for novel medications, exploration of molecular targets, and acceptance of personalized approaches through precision medicine. It is crucial to prioritize ongoing investigation into developing treatments' safety, effectiveness, and long-term results. Incorporating cutting-edge technology like AI and genomics into regular clinical procedures offers a promising opportunity to revolutionize diabetes care. This can be achieved by customizing interventions to suit the distinct attributes of each patient [20, 21]. In this remarkable scientific advancement period, fostering collaboration among researchers, doctors, and policymakers is becoming increasingly crucial. Collectively, we may mold a forthcoming era in which diabetes control is not solely efficient in managing high blood sugar levels but additionally tailored, accurate, and responsive to the varied requirements of individuals with this intricate and multifaceted ailment.

Challenges and considerations in novel therapies

With the emergence of new and advanced treatments for diabetes, it is crucial to acknowledge and address the difficulties and factors involved in putting them into practice. This essay examines three essential aspects, patient adherence and compliance, long-term safety considerations, and economic ramifications, that influence the field of innovative treatments for diabetes. To maximize patient results and ensure the long-term viability of these developments, it is essential to have a thorough grasp of the obstacles associated with these medicines since they have the potential to revolutionize treatment paradigms.

Patient Adherence and Compliance

A primary obstacle in deploying innovative diabetic treatments is patient adherence and compliance. The growing intricacy of treatment regimens, particularly with incorporating numerous innovative medications, can provide a substantial obstacle. Patients may need help managing complex drug schedules, resulting in non-compliance and less-than-ideal glucose control [21]. The prevalence of polypharmacy, which refers to the concurrent prescription of many drugs to patients, is increasing in the field of diabetes management. Although this strategy enables targeting various illness characteristics, it imposes a significant pill burden on patients. Individuals may become overwhelmed and need help adhering to their pharmaceutical regimen when they are required to manage many drugs with different dosing schedules. Patient adherence is closely connected to behavioral and lifestyle factors. Complying with drug regimens may clash with daily activities, resulting in accidental non-adherence. Furthermore, elements such as memory lapses, apprehension about potential adverse reactions, and hesitancy to incorporate drugs into daily routines can additionally lead to inadequate compliance [22]. Tackling issues with patient adherence requires embracing cutting-edge technologies, such as digital health interventions. Mobile applications and wearable gadgets can be prompts, deliver educational materials, and provide immediate feedback to promote patient involvement. Customizing interventions to suit individual tastes and needs can help reduce some of the obstacles to following new diabetic treatments [22].

Long-term Safety Concerns

Novel medicines are frequently introduced without sufficient long-term safety evidence. Clinical trials usually have a limited duration, and the actual safety characteristics of a treatment may only be fully evident once it has been used for a prolonged period. This presents difficulties in fully grasping the possible hazards linked to new substances and exceptionally uncommon or delayed adverse effects [22]. The intricate nature of biological systems gives rise to the potential for unexpected repercussions and off-target impacts when employing new therapeutic approaches. Although initial trials can offer insights into the expected effects on specific targets, the prolonged use of drugs may reveal unforeseen interactions or impacts on other physiological processes. It is crucial to have diligent post-marketing surveillance to identify and deal with any new safety concerns [23]. Individual variation in patient characteristics contributes to the variability in the safety of new treatments. Age, other medical conditions, and genetic variants might impact how people react to drugs. Hence, the task is to recognize and comprehend prospective safety issues in specific subgroups to guarantee that innovative treatments' advantages surpass any accompanying hazards [23]. Ensuring long-term safety requires prioritizing shared decision-making and securing informed consent. Healthcare professionals must actively involve patients in conversations regarding the potential dangers, advantages, and uncertainties linked to innovative treatments. By offering extensive information, patients are empowered to make well-informed decisions, ensuring that their preferences align with the potential long-term safety consequences of the selected treatment method [23].

Economic Implications

The economic consequences of new diabetic treatments are complex and have several aspects. A key factor to consider is the financial implications involved in developing, producing, and distributing these groundbreaking agents. The substantial research and development investments necessary for innovative treatments contribute to their initial exorbitant expenses, potentially constraining accessibility for specific groups of patients. The cost-effectiveness of innovative treatments is a crucial factor in determining their availability. As these agents become available in the market, concerns arise about their accessibility to a wide range of patients, regardless of their socioeconomic situation. Health inequalities may worsen if the financial cost linked to new treatments hinders vulnerable people's ability to obtain them [23]. Conversely, it is crucial to contemplate the potential economic consequences of enhanced results linked to innovative treatments. There can be subsequent economic advantages if these agents exhibit higher effectiveness, minimize problems, and improve overall health. The initial expenses of innovative therapies [23] may be compensated by a decrease in hospitalizations, a reduction in complications, and an enhancement in productivity for individuals with diabetes. Health technology assessment (HTA) is essential in determining the worth of innovative treatments. Evaluating the effectiveness of treatment in a clinical setting and its cost-efficiency enables policymakers to make well-informed decisions on reimbursement and the availability of the treatment in the market. Implementing value-based pricing models ensures that the cost of new medications is in line with their proven clinical advantages, promoting sustainability and fair access [24].

Mitigating Challenges: A Holistic Approach

Addressing difficulties related to patient adherence necessitates a comprehensive strategy that commences with patient education and empowerment. Facilitating the provision of concise and easily understandable information regarding the significance of adhering to medication, possible adverse reactions, and adjustments to one's lifestyle promotes the active involvement of patients. Enabling individuals to engage in their treatment decisions actively increases their dedication to long-term therapy. A multifaceted approach to patient care is required due to the intricacy of modern diabetic treatments. Integrating endocrinologists, primary care physicians, nurses, dietitians, and pharmacists in collaborative endeavors can effectively tackle several facets of patient management, including drug adherence and lifestyle adjustments. Coordinated care guarantees patients all-encompassing assistance in managing their treatment regimens [24]. Regularly monitoring patients receiving innovative treatments is crucial for spotting obstacles and adjusting interventions. Healthcare providers can evaluate patient progress, address emergent difficulties, and alter treatment plans by conducting regular follow-up appointments, virtual health check-ins, and utilizing technology for real-time data collection. Adopting this proactive strategy improves patient results and reduces long-term safety issues. Incorporating innovative treatments into established healthcare systems is crucial for optimizing their effectiveness. Healthcare systems must modify their structures to fit the heightened intricacy of treatment regimens by integrating support services, instructional initiatives, and efficient communication channels. An integrated system boosts patient satisfaction, facilitates compliance, and guarantees the sustained efficacy of innovative diabetic treatments [25].

Future directions and research opportunities

The dynamic field of diabetes management necessitates an ongoing examination of future directions and research opportunities to improve the effectiveness of treatments and patient outcomes. This essay explores crucial topics for more research, potential collaborations between treatments, and the consequences of forthcoming studies for diabetes recommendations. By clarifying these characteristics, we can facilitate innovations that fundamentally transform the paradigms of diabetes care.

Areas for Further Investigation

Gaining a comprehensive understanding of the complex mechanisms that form the basis of innovative diabetic treatments is crucial for improving treatment approaches. Additional examination of the molecular processes, cellular connections, and subsequent consequences of these treatments can reveal intricate insights. For instance, investigating the signaling cascades triggered by dual incretin receptor agonists or studying the effects of mitochondrial regulation on cellular metabolism could establish a basis for enhancing therapeutic approaches [26]. The advent of precision medicine calls for the investigation of personalized treatment strategies customized to the distinct attributes of every patient. Examining the interaction of genetic, metabolic, and lifestyle factors might reveal the determinants affecting the treatment response. Identifying biomarkers linked to treatment effectiveness and negative side effects can aid in creating algorithms that guide personalized therapy choices [26]. Although clinical trials offer valuable insights into the effectiveness and safety of new treatments, real-world evidence is necessary for comprehending their efficacy in various patient populations. Extended observational studies can provide insights into the long-lasting effectiveness of treatment responses, potential adverse effects occurring later, and the influence of innovative medicines in decreasing problems associated with diabetes. Gaining insights from real-world experiences is crucial for guiding clinical practice and enhancing the accuracy of treatment guidelines [26]. Exploring the incorporation of digital health solutions in diabetes treatment has great potential. Potential areas for research are evaluating the influence of mobile applications, wearable devices, and telehealth treatments on patient compliance, self-care behaviors, and medical results. Investigating the scalability and cost-effectiveness of these technologies can provide valuable insights for optimizing their inclusion into regular diabetic treatment [27].

Potential Synergies between Therapies

Exploring possible connections between new treatments provides a possibility to improve the management of blood sugar levels by using combined methods. For example, investigating the combination of GLP-1 receptor agonists with SGLT2 inhibitors may utilize complementary action techniques. The combined impact on regulating glucose levels, reducing body weight, and improving cardiovascular outcomes may provide a more holistic treatment approach for individuals with diabetes [27]. Given the diverse nature of diabetes pathogenesis, the potential exists to create tailored combination treatments. Examining the personalized reaction to particular combinations of antidiabetic medications, considering patient attributes such as age, BMI, and genetic predispositions, could result in customized treatment plans. The objective is to enhance the effectiveness of therapy while reducing the occurrence of adverse effects by employing personalized methods. The synergistic effects of medicines go beyond the combination of drugs and involve interdisciplinary teamwork. Incorporating diabetes management within comprehensive healthcare models that include endocrinologists, primary care physicians, nutritionists, and mental health practitioners can offer holistic care. Researching to investigate the effects of interdisciplinary collaboration on patient outcomes, adherence, and quality of life is crucial for designing future models of diabetes treatment [28].

Implications for Diabetes Guidelines

Ongoing research and progress in diabetes care require regular revisions to treatment algorithms in clinical guidelines. When new treatments prove effective and safe, guideline committees should include them in their recommendations for first or additional treatment options. Periodic evaluations of the evidence, collaborative decision-making among specialists, and adjustment to changing treatment environments guarantee that guidelines continue to accurately represent the most recent scientific knowledge. Investigations into personalized risk stratification can provide valuable insights for creating policies that customize treatment recommendations according to patient-specific criteria. Gaining insight into the patients most prone to deriving advantages from particular medicines or more susceptible to experiencing adverse effects enables a more sophisticated approach to making therapy suggestions. Guidelines can be updated to incorporate risk-based stratification algorithms, which can help develop personalized treatment plans [29-31]. Given the increasing importance of digital health solutions in diabetes care, it is crucial for guidelines to include suggestions about their utilization. Researching digital health initiatives' efficacy, practicality, and security can provide valuable insights for creating evidence-based recommendations. Guidelines offer helpful information on incorporating telehealth, mobile applications, and wearable devices into regular clinical practice, ensuring they align with patients' requirements and desires. Guidelines must quickly adjust to implementing new medicines, considering their distinct modes of action, safety profiles, and potential synergies. Periodic updates should encompass the dynamic nature of diabetes care, guaranteeing that healthcare providers possess the most up-to-date knowledge to make well-informed treatment choices. The guideline formulation process should be adaptable, promptly integrating current research findings and expert consensus [30-34].

## Conclusions

The progression of diabetes management goes beyond the use of drugs. It involves comprehensively comprehending patient requirements, incorporating digital health solutions, and seeking individualized, exact treatment. As we traverse this changing terrain, it becomes clear that the future of diabetes care rests not just in attaining glycemic control but also in boosting general health, minimizing complications, and improving the quality of life for those with diabetes. This shift in focus necessitates a paradigm beyond conventional metrics, encompassing a thorough and patient-centered approach to care. The knowledge obtained from this investigation pushes us toward a crucial point when the demand for more research is urgent. The areas that have been identified for further study, such as gaining a deeper understanding of how new therapies work, developing personalized treatment approaches, assessing the effectiveness of treatments in real-world settings, and incorporating digital health solutions, present an opportunity for researchers, clinicians, and policymakers to work together and influence the direction of diabetes research. The diabetes research community can contribute to a future where innovative therapies are seamlessly integrated, guidelines are dynamic and adaptive, and personalized care becomes the cornerstone of diabetes management by embracing interdisciplinary collaboration, staying attuned to patient needs, and fostering a culture of continuous learning. To summarise, achieving the best possible diabetes care is a constant effort that demands intense devotion, cooperation, and an unwavering commitment to enhancing the well-being of persons impacted by diabetes. As we begin this journey, integrating information, applying research in practical settings, and developing a patient-focused mindset serve as guiding principles, shedding light on the road ahead in the ever-changing field of diabetes care.
